# Recombinant human endostatin combined with radiotherapy promotes cardiomyocyte apoptosis in rats via TGFβ1/Smads/CTGF signaling pathway

**DOI:** 10.1186/s12872-022-02499-8

**Published:** 2022-03-12

**Authors:** Weiwei Ouyang, Shimei Fu, Xing Zhao, Shengfa Su, Jun Zhang, Daxian Luo, Lina Liu, Wenjin Ding, Dongdong Cao, Likun Liu, Zhixu He, Bing Lu

**Affiliations:** 1 Department of Thoracic Oncology, The Affiliated Hospital of Guizhou Medical University and The Affiliated Cancer Hospital of Guizhou Medical University, No. 1 Beijing Road West, Guiyang, 550004 China; 2 Stem Cell and Tissue Engineering Research Center, Guizhou Medical University, Guiyang, 550025 China; 3 Department of Pathology, The Affiliated Hospital of Guizhou Medical University and the Affiliated Cancer Hospital of Guizhou Medical University, Guiyang, 550004 China

**Keywords:** Recombinant human endostatin, Radiotherapy, Signaling pathway, Apoptosis, Lung cancer

## Abstract

**Purpose:**

The aim of the present study was to investigate the efficacy of recombinant human endostatin (ES) (rh-ES) combined with radiation on rat cardiomyocyte apoptosis and the regulatory mechanism of transforming growth factor beta1 (TGF-β1)/Sma and Mad-related protein 3 (Smad3)/connective tissue growth factor (CTGF) signaling.

**Method:**

The primary cardiomyocytes were isolated from neonatal Sprague–Dawley rats for culture in vitro and divided into blank control group (without treatment), 10 Gy radiation + siTGF-β1 siRNA (gene silencing) group, ES + siTGF-β1 siRNA group, and 10 Gy radiation + ES + siTGF-β1 siRNA group. Methyl thiazolyl tetrazolium assay was used to calculate the half-maximal inhibitory concentration (IC_50_) of rh-ES on cardiomyocytes. Adenoviral vector was constructed for virus packaging to silence TGF-β1 expression in cardiomyocytes. Quantitative real-time polymerase chain reaction and Western blot were carried out to analyze TGF-β1, Smad2, Smad3 and CTGF expression at both gene and protein levels. Flow cytometry and electron microscope were used to examine cell apoptosis.

**Results:**

ES had a dose-dependent inhibitory effect on the proliferation of primary rat cardiomyocytes. ES combined with radiotherapy significantly inhibited cardiomyocyte proliferation and promoted cell apoptosis (*P* < 0.01). The gene and protein expression of TGF-β1, Smad2, Smad3 and CTGF were significantly up-regulated in primary cardiomyocytes transfected with TGF-β1 gene (*P* < 0.05).

**Conclusion:**

The combination therapy with rh-ES and radiation can promote cardiomyocyte apoptosis and aggravate myocardial cell damage via TGF-β1/Smad3/CTGF signaling pathway.

**Supplementary information:**

The online version contains supplementary material available at 10.1186/s12872-022-02499-8.

## Introduction

Loss of cardiomyocytes occurs with aging and contributes to cardiovascular complications. As the rate and amount of cardiomyocyte loss is the most important determinant of patient morbidity and mortality, novel treatment strategies targeting apoptosis are crucial [1]. Radiotherapy has a wide range of adaptability with a relatively great curative effect. However, Zhu et al. evaluated the effect of microwave radiation on rat cardiomyocyte apoptosis and found that cardiomyocyte apoptosis was increased in a dose-dependent manner after microwave radiation [2]. Another animal model study also demonstrated that radiation could lead to cardiomyocyte apoptosis [3]. Moreover, when patients with malignant tumor receive radiotherapy, it is inevitable for the heart to receive a certain dose of radiation that may cause cardiac injury [4, 5].

Apart from radiation, endostatin (ES), the first-in-class recombinant human ES (rh-ES) anti-cancer drug developed by Chinese researchers, has been widely used in the treatment of advanced lung cancer [6]. ES combined with radiotherapy can significantly prolong the survival time of advanced non-small cell lung cancer (NSCLC) patients [7]. However, cardiotoxicity is still the major adverse effect of rh-ES. In the phase I/II clinical trials of ES, 6.38% (30/470) of advanced NSCLC patients treated with ES developed adverse cardiac reactions [8]. ES in combination with radiotherapy may directly affect physiological dysfunction of cardiomyocytes, resulting in increased cardiotoxicity, which has been reported in the phase I to III clinical trials conducted at home and abroad [9]. At present, there is no research to explore the intrinsic mechanism and interrelationship of the two combined therapies.

Transforming growth factors-β1 (TGF-β1) is a multipotent cytokine that can promote cell proliferation and differentiation. In myocardial fibrosis, TGF-β1 is considered to be the strongest fibrogenic growth factor [7], which can promote collagen synthesis and inhibit degradation by activating downstream Smad proteins, resulting in an imbalance of synthesis and degradation, thereby leading to myocardial fibrosis. In addition, it can induce the expression of connective tissue growth factor (CTGF) [10]. Effects of ES combined with radiotherapy on myocardial injury and fibrosis, as well as the differences in gene and protein expression of TGF-β1/Smad/CTGF signaling pathway have been reported [11]. Whereas the relationship between TGF-β1/Smad signaling pathway and apoptosis of rat cardiomyocytes induced by rh-ES combined with radiotherapy has not been systematically studied.

In this study, we examined the effects of radiotherapy combined with ES on cardiomyocytes and the potential mechanisms, providing additional references for the treatment of cardiac injury.

## Material and methods

### Experimental grouping

Primary cardiomyocytes isolated from 20 neonatal Sprague–Dawley rats (Animal Experiment Centre of Guizhou Medical University) were randomly divided into blank control group, 10 Gy radiation + siTGF-β1 group, ES + siTGF-β1 group, and 10 Gy radiation + ES + siTGF-β1 group, with 5 rats in each group. The cells in control group were cultured normally without any treatment, while the cells in experimental groups were transfected with virus, followed by treatment with 529 μg/ml ES (Simcere Bio-Pharmaceutical Co., Ltd, Shandong) (Additional file 1: Figure S1). Irradiation modality (10 Gy radiation (Elekta Synergy, Stockholm, Sweden)): source skin distance 1 m, basal area for irradiation 106.5 cm^2^ and irradiation depth 2.22 cm. These treatments were administered in parallel. This study was approved by the Ethics Committee of the Affiliated Hospital of Guizhou Medical University (SYXK 2012–001).

### Isolation and primary culture of cardiomyocytes

Newborn Sprague–Dawley rats (1–3 days after birth) were sacrificed by cervical dislocation. Tissues were taken out from the apex of thoracic cavity and cut into particles of 1 mm^3^, followed by rinse in phosphate buffered saline (PBS) containing double-antibody. After centrifugation, the supernatant was collected and filtered through 200 mesh cell sieves, and the filtrate was centrifuged at 300 × g for 5 min. Then supernatant was discarded, and cell sedimentation was preserved for later use [12].

Rat cardiomyocytes were suspended in complete culture medium and inoculated into flask A, followed by incubation at 37 °C in a 5% CO_2_ constant temperature incubator for 30 min. Non-adherent cells were aspirated to inoculate on flask B, and placed in 5% CO_2_ incubator for static culture at 37 °C. Subsequently, Cytarabine 2 μg/mL was added to flask A 24 h later, and the cells were cultured for another 48 h. Finally, the medium was changed to normal complete medium for rat cardiomyocytes and culture for 3–5 days.

### Morphological study of primary cardiomyocytes

The freshly isolated cardiomyocytes were uniformly suspended in a circular medium under a phase contrast microscope. The cells began to adhere to the walls after 2 days of culture. The morphological changes of myocardial cells were observed via an inverted phase contrast microscope and video (Olympus CKX53; Olympus Sales Service Co., Ltd, Beijing). Most cardiomyocytes grew from round to spindle cells and spread gradually. Some cells were polygonal or irregular in shape with extending pseudopodia. Meanwhile, individual adherent cells showed spontaneous beating at inconsistent frequencies. The pseudopodia of some cells touched each other to form cell clusters after culture for 4 days. The cells showed synchronized spontaneous beating. The cell viability now was optimal for subsequent experiments.

### Immunofluorescence

The sliced cardiomyocytes were taken and fixed with 40 g/L paraformaldehyde for 10 min. After washing 3 times with PBS, 0.03% Triton X 100 (Amresco, WA, USA) was added and incubated for 30 min, followed by a blockage by 5% Goat serum (Thermo Fisher Scientific, USA) at room temperature for 30 min. Then 1:100 diluted rat anti-mouse alpha-sarcomeric actin (α-SCA) antibody (Santa Cruz, sc-58670) was added and incubated at 4 °C overnight (negative control was replaced by PBS), followed by incubation with 1:100 diluted goat anti-rabbit IgG antibody (Invitrogen, Carlsbad, CA) for 1.5 h. 4',6-diamidino-2-phenylindole (DAPI) (Invitrogen, Carlsbad, CA) was used to cover the film, and sections were visualized under an immunofluorescence microscope, following calculation of cardiomyocytes purity [13].

### Methyl thiazolyl tetrazolium (MTT) assay

After 4 days of primary rat cardiomyocytes culture, ES was added to the culture media to make the final concentration of ES to 12.5, 25, 50, 100, 200, 400 and 600 μg/ml, respectively. Cells were cultured at 37 °C for 24 h. Each group was set with 3 replicates. After addition of 10 μl Methyl thiazolyl tetrazolium (MTT, Solarbio, Beijing Soleibao Technology Co., Ltd) to each well, the cells were cultured at 37 °C for another 4 h, and the culture media was discarded. Then 150 μl dimethyl sulfoxide was added and shaken for 10 min. The optical density (OD) of each well was measured at 568 nm with an enzyme-linked immunosorbent assay (ELISA) microplate reader [14]. MTT data were used to construct Morgan–Morgan–Finney mathematical model with Curve Expert software (version 1.4) for calculating the IC_50_ of ES on primary rat cardiomyocytes.

### TGF-β1 siRNA screening

TGF-β1 siRNA fragments were synthesized by GenePharma (Shanghai, China). Three siRNA fragments targeting rat TGF-β1 and one negative control (NC) fragment (green fluorescence) were shown as follow [15]. The negative control group sense (5’-3’) and antisense (5’-3’) sequence was UUCUCCGAACGUGUCACGUTT and ACGUGACACGUUCGGAGAATT, respectively. The TGF-β1-rat-520 group sense (5’-3’) and antisense (5’-3’) sequence was AGACAUCACACACAGUAUATT and UAUACUGUGUGUGAUGUCUTT, respectively. The TGF-β1 siRNA-811 group sense (5’-3’) and antisense (5’-3’) sequence was CUGCUCUUGUGACAGCAAAGA and UCUUUGCUGUCACAAGAGCAG, respectively. The TGF-β1 siRNA-1000 group sense (5’-3’) and antisense (5’-3’) sequence was CUUCAGCUCCACAGAGAAGAA and UUCUUCUCUGUGGAGCUGAAG, respectively. All transfections were performed using Lipofectamine 2000 reagent (11,668–019; Invitrogen Inc., Carlsbad, CA) according to the manufacturer's instructions. Cells were collected at 24 h after transfection for microscopic examination. Q-PCR assay was carried out to confirm the efficiency of interference.

### Adenovirus packaging

Plasmid vector pDC316-ZsGreen-shRNA was confirmed by restriction enzyme *XhoI* and DNA sequencing analysis. On the day before transfection, cells were inoculated in a 6-well plate at a density of 3–5 × 10^5^ cells per well. When the fusion degree of cells was about 70%, transfection was carried out via Lipofectamine 2000. After 4–6 h of culture, the culture medium was changed and continued in 5% CO_2_ incubator at 37 °C. After the cells grow into the culture dish, the cells were subcultured in a 25 cm^2^ cell culture dish. When the cells grow to the bottom of the bottle, cells were transferred into a 75cm^2^ cell culture bottle to observe the signs of toxicity. Virus, the first generation of P1, was collected when most of diseased cells fell off the culture bottle wall. When the fusion degree reached 80%-90%, P1 generation virus was added. Two days after infection, the cells were collected, frozen and thawed at -80 °C/37 °C for 3 times, followed by centrifugation at 12,000 × g for 10 min, amplification to P3 generation and purification with Virabind adenovirus purification kit (Cell Biolabs, CA, USA) [16]. The titer of adenovirus was determined.

### Quantitative real-time PCR

According to the manufacturer's instructions, total RNA was extracted with Trizol reagent (Aidlab, Beijing, China) [17]. Briefly, the cells were cleaved to 1 mltrizol, then total RNA was extracted with 200 μl chloroform, and RNA was precipitated with 400 μl isopropyl alcohol. The total RNA was reverse-transcribed to cDNA with hiscript reverse transcriptase (vazyme, Nanjing, China). The cDNA was diluted 7 times with ddH_2_O (Genecopoeia, C1D230A), and the amplification and detection were performed by real-time quantitative using SYBR Green Master Mix (Vazyme, Nanjing, China) in QuantStudio 6 real-time PCR system (Dongsheng innovation Biotechnology, Beijing, China). Data were analyzed using the 2^−ΔΔCt^ method and was normalized by glyceraldehyde-3-phosphate dehydrogenase (GAPDH) expression in each sample. The primer sequences of the study were listed in Table 1.

**Table 1:** Primers list

Name	Primer	Sequence	Size
Rat GAPDH	Forward	5'-ACAGCAACAGGGTGGTGGAC-3'	253 bp
	Reverse	5'-TTTGAGGGTGCAGCGAACTT-3'	
Rat TGFβ1	Forward	5'-GTGGCTGAACCAAGGAGACGGAATA-3'	118 bp
	Reverse	5'-ACCTCGACGTTTGGGACTGATC-3'	
Rat CTGF	Forward	5'-GAAATGCTGTGAGGAGTGGGTGTGT-3'	123 bp
	Reverse	5'-CAGTTGGCTCGCATCATAGTTGGGT-3'	
Rat smad2	Forward	5'-GGCTGAACTGTCTCCTACCACTCTC-3'	289 bp
	Reverse	5'-ACCTATGTAATACAAGCGCACTCCC-3'	
Rat smad3	Forward	5'-CCTCTCCCCGAATCCGATGTCC-3'	259 bp
	Reverse	5'-CCTCCCAATGTGCCGCCTTGTA-3'	

### Flow cytometry

Cells were collected after digestion with 0.25% trypsin containing EDTA, washed twice with PBS, and suspended in binding buffer (500 μl). Then cell suspension was stained with Annexin V-FITC 5 μl and propidium iodide 5 μl for 5–15 min at room temperature [18]. The apoptotic rate was measured by flow cytometry (Gallios, Beckman Coulter, Brea, CA).

### Western blot analysis

Total proteins were extracted by lysis buffer. Samples (30 μg protein/lane) were separated by sodium dodecyl sulfate polyacrylamide gel electrophoresis and transferred onto poly vinylidene fluoride (PVDF) membranes (0.22 µm pore, Roche), which were then blocked with 5% skim milk. Subsequently, membranes were incubated overnight at 4 °C with primary antibodies of GAPDH, anti-BCL-2 associated X (Bax), anti-Bcl-2, anti-Capase3, anti-P53, rabbit polyclonal antibody to TGF-β1, rabbit polyclonal antibody to CTGF and rabbit polyclonal antibody to Smad2/3 (Abcam, UK), respectively. Then, the membranes were incubated with secondary antibody for 1 h at room temperature. Enhanced chemiluminescence enhancer was mixed well with HRP solution (Millipore, Burlington, MA, USA) in a ratio of 1:1. The working solution was spiked onto PVDF membrane, and the excess substrate solution was removed for several minutes. The proteins were scanned and analyzed with BandScan software to obtain the grayscale value. The OD value of the protein sample was measured on DG-3022 Aplate reader at 568 nm, and the protein content in the sample was calculated by regression equation [19].

### Structural change of cells under transmission electron microscope (TEM)

The cell mass or tissue was fixed with 2.5% glutaraldehyde and 0.1 M PBS (PH7.4) for 2–4 h at 4 °C. The samples were rinsed with 0.1 M PBS for 3 times (15 min each). The samples were fixed with 1%osmic acid and 0.1 M PBS (pH 7.4) at room temperature (20 °C) for 2 h. The rinsing process was repeated with 0.1 M PBS (pH 7.4) for 3 times (15 min each). The samples were dehydrated with alcohol after the above procedures, followed by impregnation with pure 812 embedding agent, embedding, section, and uranyl-lead double staining (2% uranium acetatesaturated aqueous solution and lead citrate, 15 min each). The sections were dried at room temperature overnight and observed by TEM (H-7650; HITACHI) [18].

### Statistical analysis

The data were expressed as mean ± standard deviation. SPSS software (version 21) was used for statistical analysis. One-way analysis of variance was used to compare the difference between multiple groups. *P* < 0.05 was defined as statistical significance.

## Results

### TGF-β1 interference screening and adenovirus packaging

**Fig. 1:**
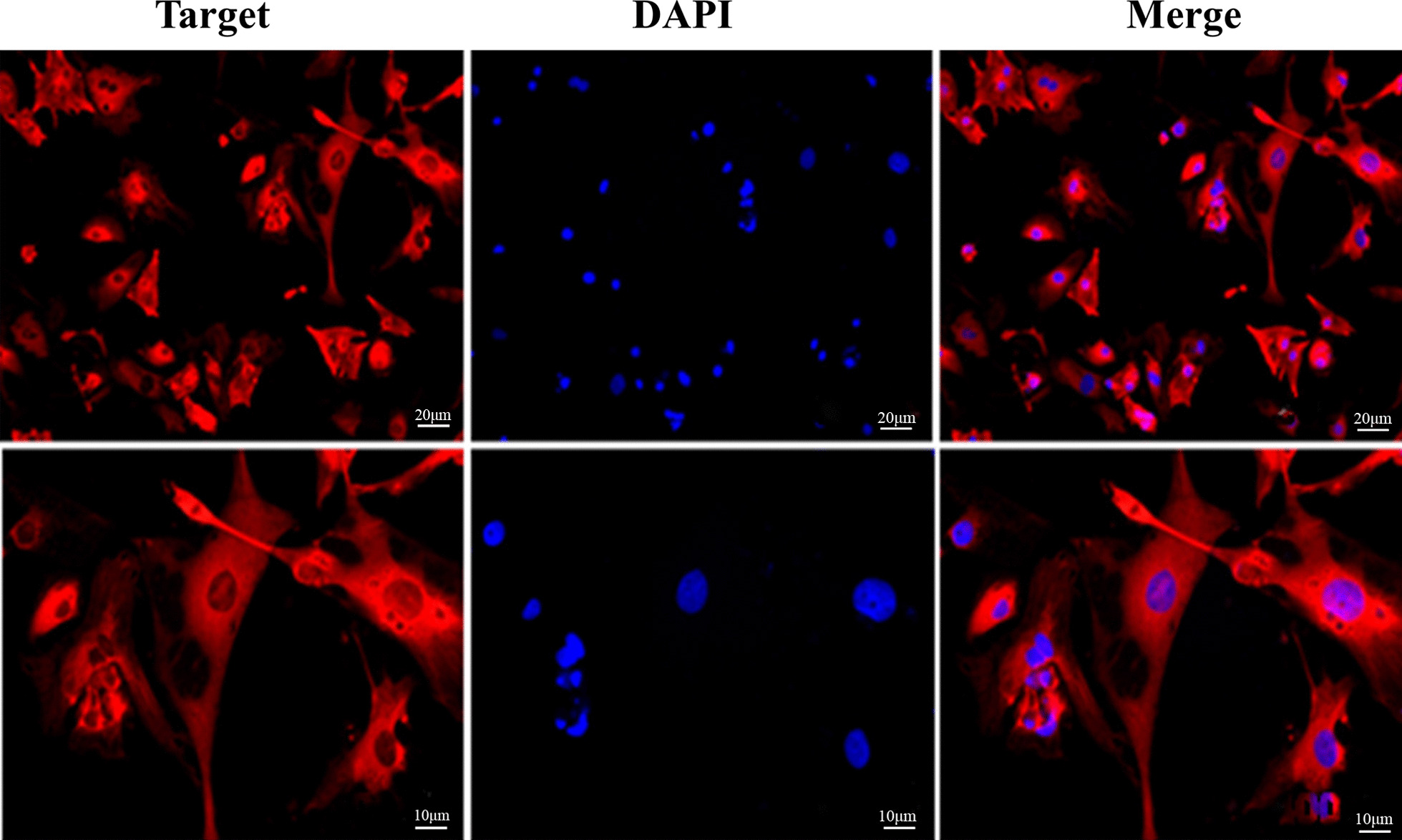
Expression of α-Sca. After 4 days of culture, the cardiomyocytes were seen under fluorescence microscope, with DAPI staining of nuclei (blue fluorescence) and labeled α-SCA protein (red fluorescence)

The positive rate of α-SCA expression was higher than 98% in cytoplasm (Fig. 1). MTT results showed that treatment with ES (12.5, 25, 50, 100, 200, 400 and 600 μg/ml) suppressed the proliferation of primary rat cardiomyocytes in a dose-dependent manner. The IC_50_ of ES was 529 μg/ml at 24 h. Therefore, ES 529 μg/ml was selected for subsequent experiments.

**Fig. 2:**
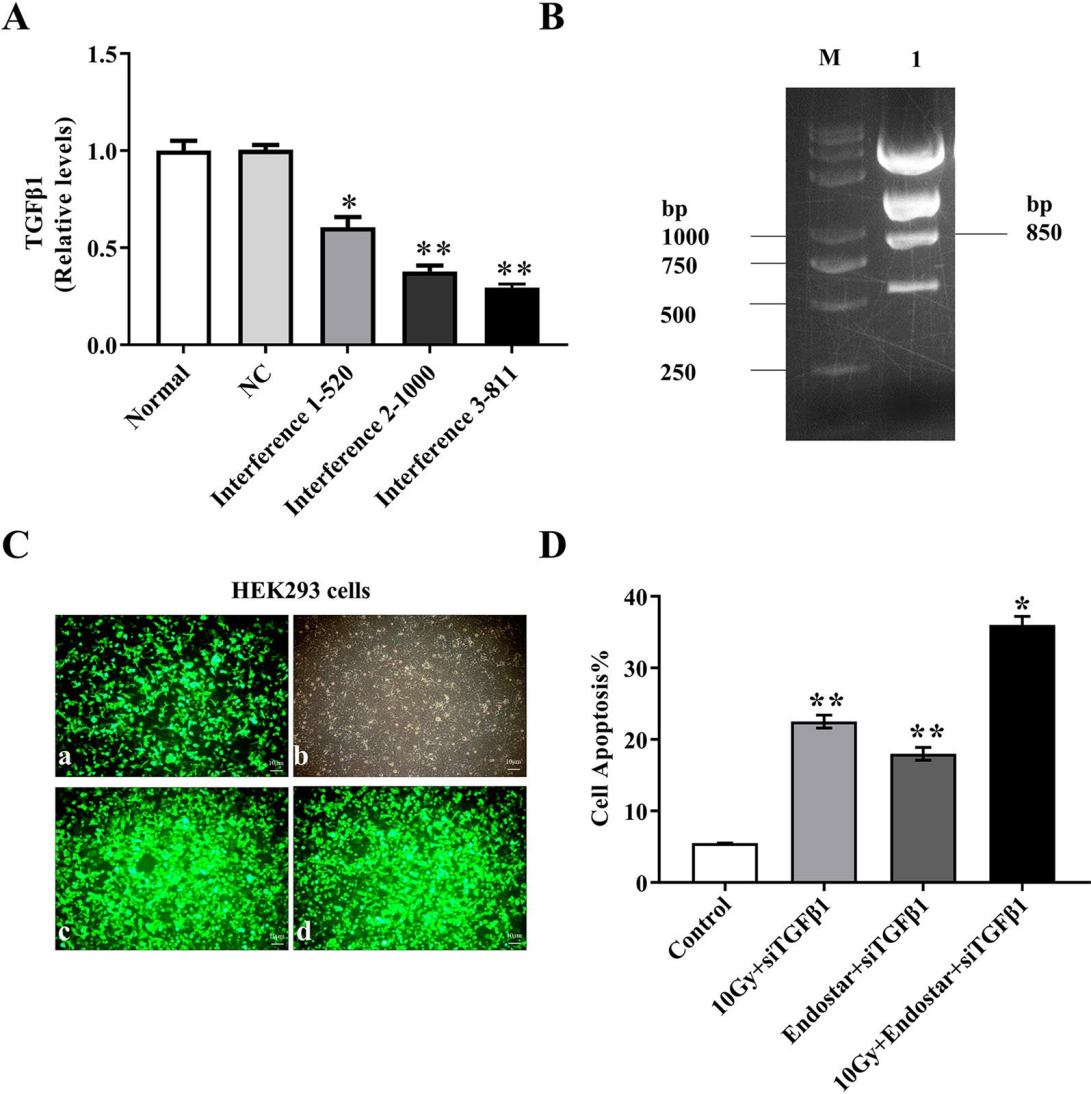
**A** TGF-β1 expression level after interference; **B** Positive clone results; **C a**-**b** HEK293 was transfected with plasmid pDc316-Zsgreen-rTGFβ1 siRNA-811, **c**-**d** HEK293 cells were transfected with adenovirus Zsgreen-rTGFβ1 siRNA-811; **D** Effects of different treatments on apoptosis rate of rat cardiomyocytes (n = 5). *P < 0.05 compared with the control group, **P < 0.01

The results of TGF-β1 interference efficiency demonstrated good amplification curves of internal reference gene GAPDH and target gene TGF-β1 with a single peak of melting curve, proving good performance of the primers. TGF-β1 expression (0.294) after treatment with siRNA fragment 3–811 was lower than that of normal group and NC group. Fragment 3–811 had the best interference efficiency (70.6%) among the three fragments (Fig. 2A, *P* < 0.01). The recombinant adenovirus plasmid was digested with *XhoI* to obtain 850 bp recombinant adenovirus. From Fig. 2B, the positive clone was pDC316-ZsGreen-rTGFB1 siRNA-811–1#, and the recombinant adenovirus vector was constructed successfully. The positive clones were digested and identified as pDC316-ZsGreen-rTGFB1 siRNA-811–1#, and the sequencing results were consistent with the designed sequence. The number of fluorescent cells decreased with the passage of transfected cells, but 6–10 days later, the number of fluorescent cells increased gradually to cell mass and patches of cells on day 12 (Fig. 2C). Human Embryonic Kidney 293 (HEK293) infected with AD ZsGreen shRNA and ad-zsgreen rTGF-β1 sirna-811 showed green under fluorescence microscope, with different green fluorescence intensity of each cell, indicating that the constructed adenovirus vector could express exogenous gene in cardiomyocytes (Fig. 2C).

### Effect of different treatments on rat cardiomyocytes apoptosis

Compared with the control group, the apoptosis rate and the expression of apoptotic factors P53, Caspase and Bax were increased significantly in three treatment groups, in contrast to a significant decrease in Bcl-2 expression, among which the apoptosis rate of 10 Gy radiation + ES + siTGFβ1 group was 35.12 ± 1.23%, almost 7 times of the control group (Fig. 2D, Additional file 1: Figure S2, *P* < 0.01).

**Fig. 3:**
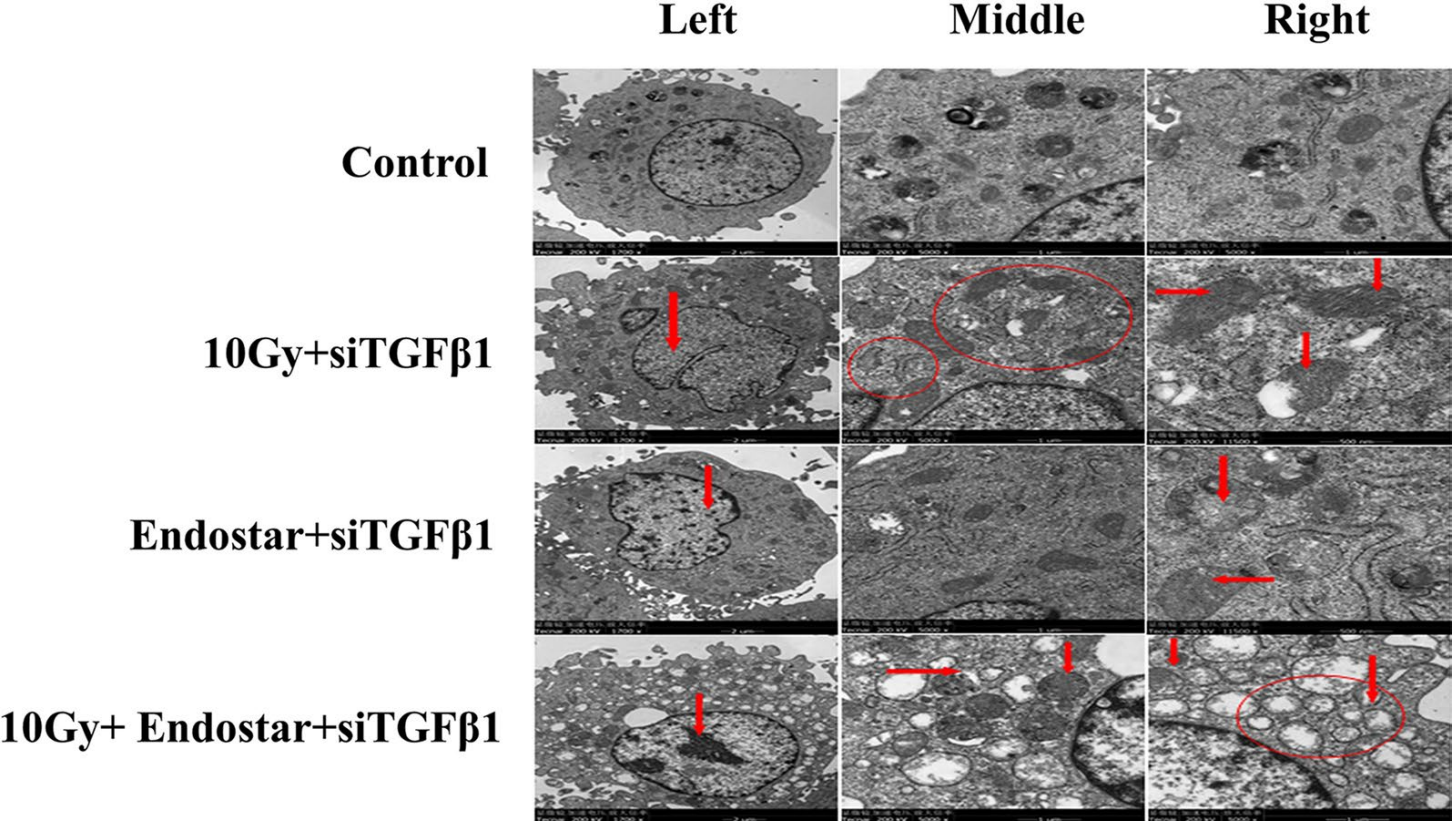
Structural changes of rat cardiomyocytes after different treatments as observed under TEM (n=5). Normal structure of nucleusand some autophagosomes in the cytoplasm just as in normal cardiomyocytes; aberration of nucleus after radiation indicated by red arrow in the leftpane; mitochondrial apoptosisseen in the cell as shown by the red circle and arrow in the middle and right panes; ES treatment resulted in chromatin shrinkage and condensed as black starry spots in nucleus indicated by red arrow in the left pane; mitochondrial apoptosis and some autophagosomes (indicated by red arrow in right pane) seen in the cell; radiation + ES combination treatment resulted in large mass of chromatin clot in the nucleus, serious structural damage of intracellular organelles, and many vacuole structures

Morphological and ultra-structural characteristics of apoptosis after different treatments were examined by TEM (Fig. 3). Results showed that the nuclear structure of the control group was normal and there was autophagy in the cytoplasm, which was the same as that of normal cardiomyocytes. In the 10 Gy radiation + siTGF-β1 siRNA, aberration of nucleus after radiation indicated by red arrow in the left pane; mitochondrial apoptosis seen in the cell as shown by the red circle and arrow in the middle and right panes; In the ES + siTGF-β1 siRNA, ES treatment resulted in chromatin shrinkage and condensed as black starry spots in nucleus indicated by red arrow in the left pane; mitochondrial apoptosis and some autophagosomes (indicated by red arrow in right pane) were seen in the cell; In the 10 Gy radiation + ES + siTGF-β1 siRNA, radiotherapy and ES combination treatment resulted in large mass of chromatin clot in the nucleus, serious structural damage of intracellular organelles, and many vacuole structures, which may be apoptotic mitochondria and autophagosomes.

### The expression levels of TGF-β1, Smad2, Smad3 and CTGF were detected by Q-PCR and Western blot

**Fig. 4:**
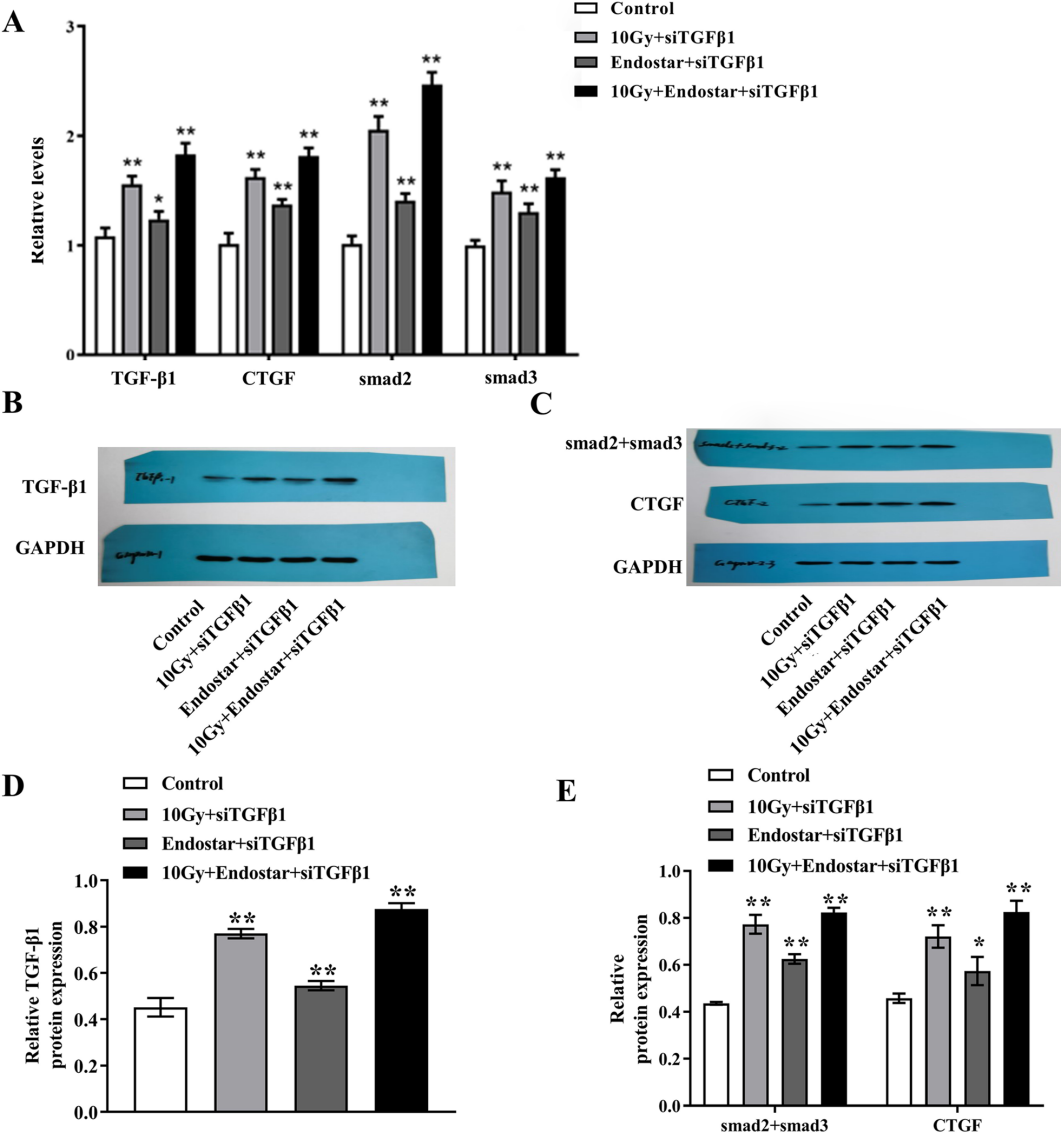
The expression levels of TGF-β1 (**a**, **b**, **d**), Smad2, Smad3 and CTGF (**c–e**) were detected by Western blot and qRT-PCR (n = 5). Western blot results were cropped and the original, uncropped gels or blots were provided in the supplemental file (Additional file 1). *P < 0.05 compared with the control group, **P < 0.01

We next explored the potential mechanism that resulted in the superior efficacy of ES combined with radiotherapy. The results clarified that the gene and protein expressions of TGF-β1, CTGF and Smad2/3 were up-regulated in primary cardiomyocytes transfected with TGF-β1 gene, which were significantly higher than those in untreated or gene silenced cells (Fig. 4, *P* < 0.05). These data indicated that ES in combination with radiotherapy could induce cardiomyocyte apoptosis through TGF-β1/Smads/CTGF signaling pathway.

## Discussion

In recent years, radiotherapy and targeted therapy have played an important part in cancer treatment. However, radiation-induced heart disease has become a typical cause of noncancerous death in patients with malignant lymphoma, breast cancer or thoracic cancer after radiotherapy. Additionally, radiotherapy not only damages vascular endothelial cells, leading to ischemic heart disease but also directly damages cardiomyocytes. Zhang et al. have found that irradiation can lead to apoptosis of H9C2 cardiomyocytes [20]. ES combined with radiotherapy aggravated myocardial fibrosis after radiation through TGF-β1/Smad3/CTGF signaling pathway [11]. In this study, we found that the combined treatment promoted rat cardiomyocyte apoptosis via TGF-β1/Smad3/CTGF signaling pathway. In this study, we found that the inhibitory effect of ES on cardiomyocytes was exacerbated after 10 Gy radiotherapy. It was shown that low doses of radiation can prevent diabetes-induced heart disease by activating the protein kinase B (AKT) pathway to improve cardiac function and hypertrophy [21]. However, finding the threshold of dose to inhibit ES-induced apoptosis in cardiomyocytes still requires further studies.

ES is a stable targeted anti-angiogenic drug with longer half-life [22]. It has been proved that ES is effective against malignant tumors such as lung cancer [23–25], but there are some adverse reactions, especially cardiotoxicity. Xu et al. found that the addition of ES to chemotherapy regimens such as FOLFOX4 resulted in higher objective response rates and longer time to disease progression, as well as the development of cardiotoxic reactions [26]. And cardiomyocyte mitochondria may be the target of ES cardiotoxicity [27]. Chen et al. showed that ES combined with radiotherapy significantly inhibited tumor growth and downregulated the expression of TGF-β1 and inflammatory and immune factors [28]. Consistently, in our study, we found that both radiotherapy and ES alone or in combination promoted apoptosis in cardiomyocytes, but the combination treatment was the most effective. In addition, ES may cause obvious pathological changes and ultrastructural damage, significantly promoting cardiomyocyte apoptosis in a dose-dependent manner. Studies have shown that ES may inactivate the Akt pathway in a time- and dose-dependent manner, significantly inhibiting the proliferation of human lung squamous carcinoma cells and enhancing the sensitivity of lung cancer cells to radiation [29, 30]. However, there is no evidence that the Akt pathway plays an effective role in the induction of apoptosis in cardiomyocytes by ES combined with radiotherapy.

We further investigated the mechanism of ES combined with radiotherapy in cardiomyocytes and showed that ES combined with radiotherapy induced apoptosis through the TGF-β1-Smad signaling pathway. TGF-β1 is a vital pro-fibrogenic factor belonging to the newly identified TGF-β1 superfamily, which is implicated in the regulation of cell growth and differentiation. However, the role of TGF-β1 in apoptosis has been variously described. Contrary to our results, the Wang and colleagues showed that TGF-β prevented anoxia-reoxygenation-induced apoptosis and improved myocardial function in rat hearts injured by ischemia–reperfusion [31]. The exact reasons for this need further research to be discovered. Moreover, the function of TGF-β1 on cell apoptosis is closely related to cell type and cell niche [32]. Dunker believed that TGF-β1-induced apoptosis played an important role in the development of limbs [33]. Smads protein is a downstream signaling molecule of TGF-β family, and is the only substrate of TGF-β1. Myocardial fibrosis is associated with up-regulation of TGF-β1 and the downstream Smad2 and Smad3 proteins, as well as down-regulation of Smad7 expression [34]. In our results, after transfection of primary cardiomyocytes with TGF-β1 gene silencing, the gene and protein expression of TGF-β1, CTGF and Smad2/3 in 10 Gy radiation + ES + siTGF-β1 siRNA group was remarkably higher than those of radiotherapy or ES alone, indicating that ES in combination with radiotherapy worsened cardiomyocyte apoptosis. We speculate that the TGFβ1/Smads/CTGF signaling pathway may participates in cardiac fibrosis and remodeling by regulating downstream CTGF gene. However, this needs to be verified in a follow-up study.

## Conclusion

Rradiotherapy with ES may aggravate myocardial cell injury more seriously than 10 Gy radiation or ES treatment alone. Moreover, the combination treatment-induced myocardial cell injury may be related to TGFβ1/Smads/CTGF signaling pathway. Moreover, this study suggests that the combination therapy of ES and radiotherapy should be avoided in patients at critical dose.

## Supplementary information

**Additional file 1. Figure S1.** Flow chart. **Figure S2.** Expression of apoptosis-related factors.

## Data Availability

The datasets used and/or analysed during the current study are available from the corresponding author on reasonable request. Data sharing is not applicable to this article as no datasets were generated or analysed during the current study.

## Abbreviations

α-SCA: Alpha-sarcomeric actin
AKT: Protein kinase B
CTGF: Connective tissue growth factor
DAPI: 4',6-Diamidino-2-phenylindole
ES: Endostatin
ELISA: Enzyme-linked immunosorbent assay
GAPDH: Glyceraldehyde-3-phosphate dehydrogenase
HEK293: Human Embryonic Kidney 293
MTT: Methyl thiazolyl tetrazolium
NC: Negative control
NSCLC: Non-small cell lung cancer
OD: Optical density
PBS: Phosphate buffered saline
PVDF: Poly vinylidene fluoride
rh-ES: Recombinant human ES
TGF-β1: Transforming growth factors-β1
TEM: Transmission electron microscope
