# Supplementary material for: Recombinant human endostatin combined with radiotherapy promotes cardiomyocyte apoptosis in rats via TGFβ1/Smads/CTGF signaling pathway
Source: BMC Cardiovasc Disord. 2022 Mar 12;22:97. doi: 10.1186/s12872-022-02499-8 (PMC8917752; doi:10.1186/s12872-022-02499-8)
Supplement: Supplementary file 1 — Additional file 1. Figure S1. Flow chart. Figure S2. Expression of apoptosis-related factors. [file 12872_2022_2499_MOESM1_ESM.zip › 12872_2022_2499_MOESM1_ESM/12872_2022_2499_MOESM1_ESM/Supplementary figure legends.docx]

Figure S1. Flow chart

Figure S2. Expression of apoptosis-related factors

Western blot results were cropped and the original, uncropped gels or blots were provided in the supplemental file. ^*^*P*<0.05 compared with the control group, ^**^*P*<0.01, ^***^*P*<0.001.
