# Supplementary figures and images for: Recombinant human endostatin combined with radiotherapy promotes cardiomyocyte apoptosis in rats via TGFβ1/Smads/CTGF signaling pathway
Source: BMC Cardiovasc Disord. 2022 Mar 12;22:97. doi: 10.1186/s12872-022-02499-8 (PMC8917752; doi:10.1186/s12872-022-02499-8)

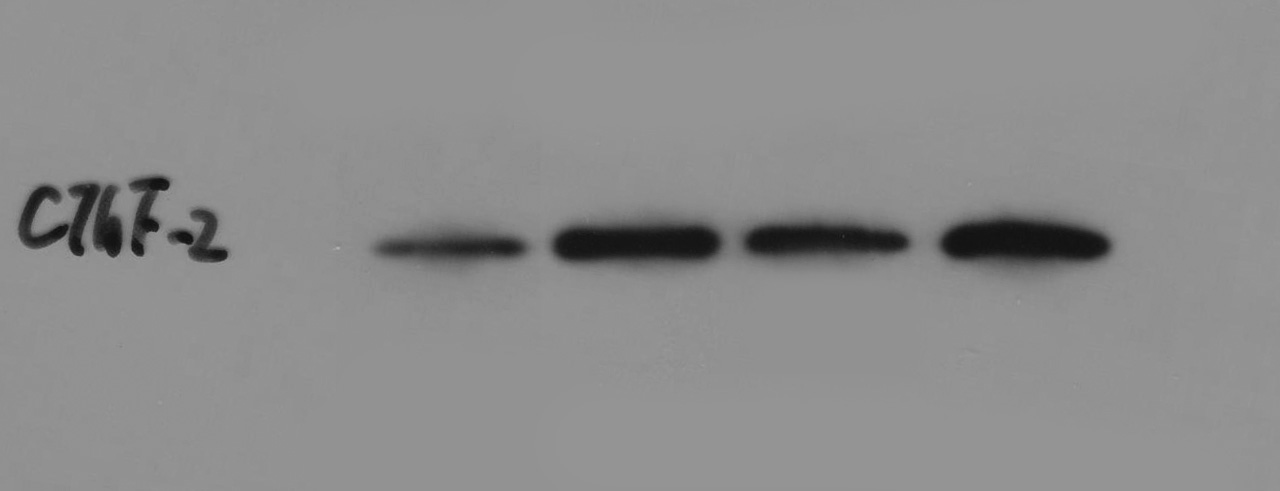

Supplement: Supplementary file 1 — Additional file 1. Figure S1. Flow chart. Figure S2. Expression of apoptosis-related factors. [file 12872_2022_2499_MOESM1_ESM.zip › 12872_2022_2499_MOESM1_ESM/12872_2022_2499_MOESM1_ESM/CTGF-2.jpg]

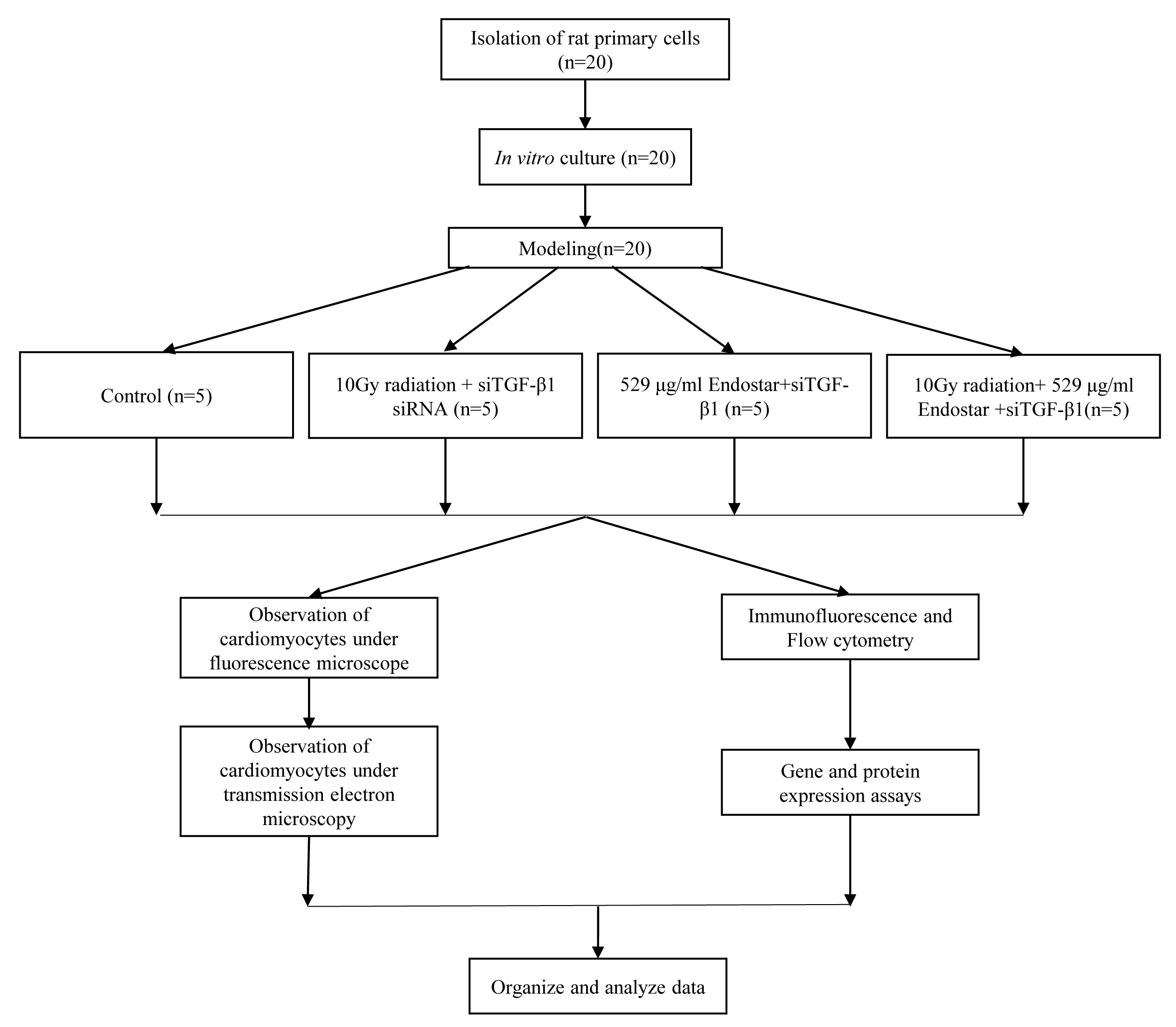

Supplement: Supplementary file 1 — Additional file 1. Figure S1. Flow chart. Figure S2. Expression of apoptosis-related factors. [file 12872_2022_2499_MOESM1_ESM.zip › 12872_2022_2499_MOESM1_ESM/12872_2022_2499_MOESM1_ESM/Figure S1.tif]

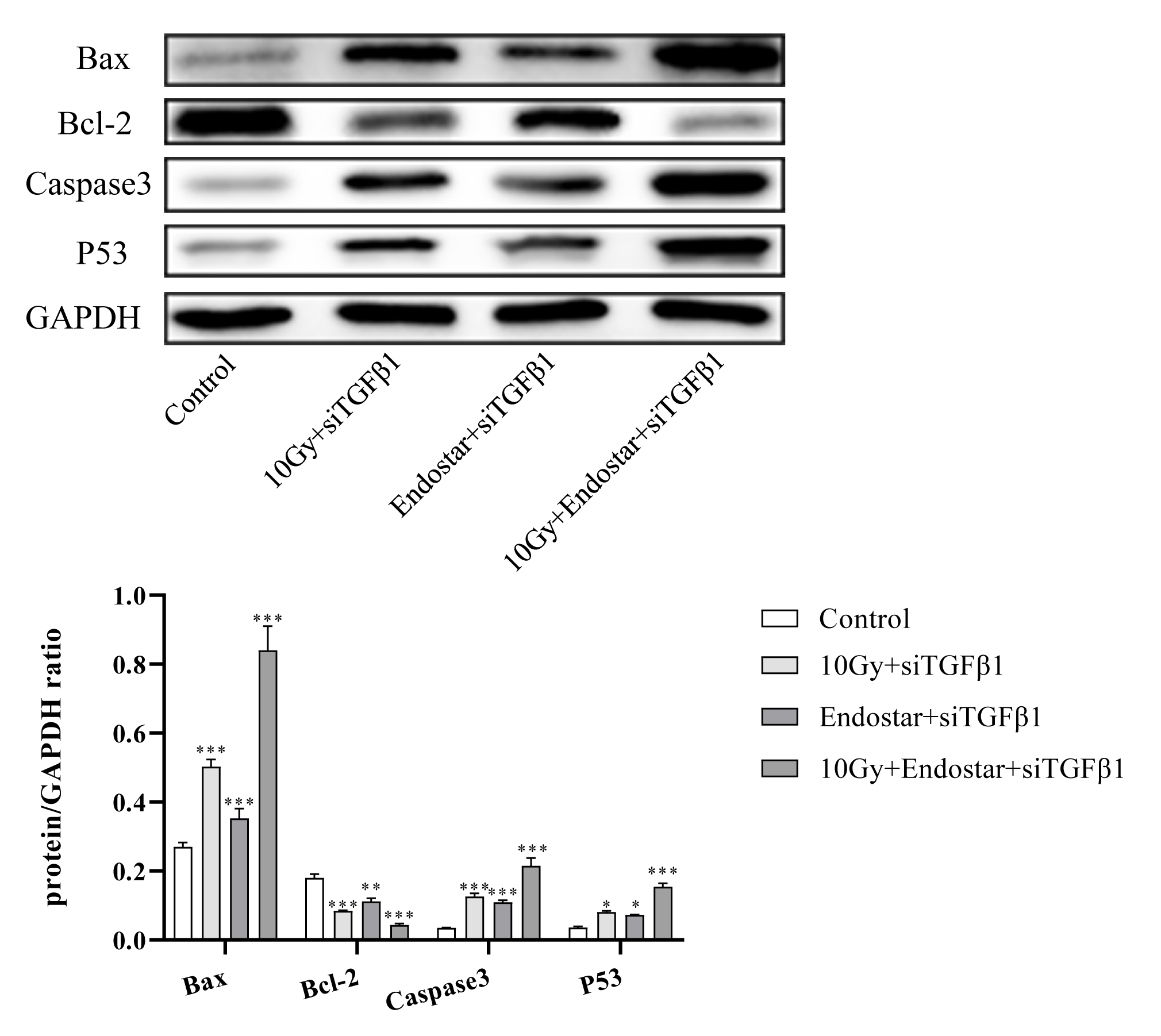

Supplement: Supplementary file 1 — Additional file 1. Figure S1. Flow chart. Figure S2. Expression of apoptosis-related factors. [file 12872_2022_2499_MOESM1_ESM.zip › 12872_2022_2499_MOESM1_ESM/12872_2022_2499_MOESM1_ESM/Figure S2.tif]

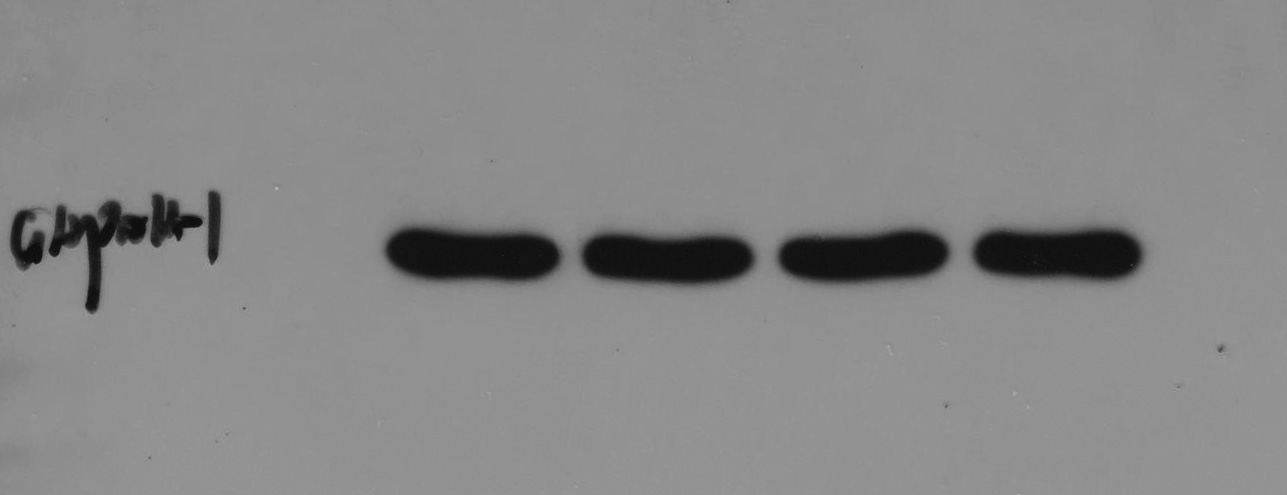

Supplement: Supplementary file 1 — Additional file 1. Figure S1. Flow chart. Figure S2. Expression of apoptosis-related factors. [file 12872_2022_2499_MOESM1_ESM.zip › 12872_2022_2499_MOESM1_ESM/12872_2022_2499_MOESM1_ESM/GAPDH (Figure4 B).jpg]

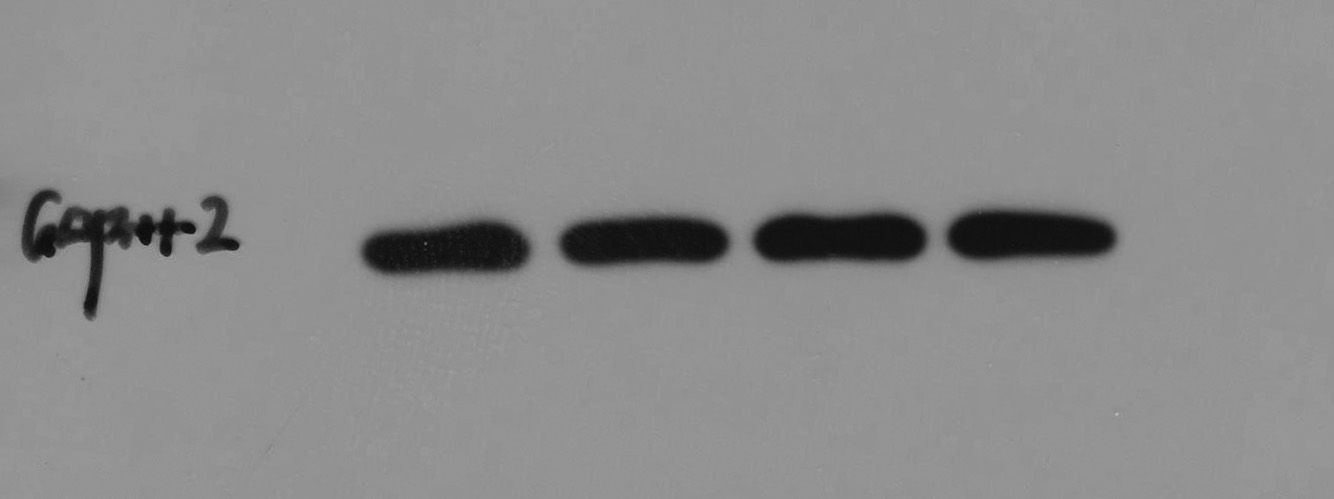

Supplement: Supplementary file 1 — Additional file 1. Figure S1. Flow chart. Figure S2. Expression of apoptosis-related factors. [file 12872_2022_2499_MOESM1_ESM.zip › 12872_2022_2499_MOESM1_ESM/12872_2022_2499_MOESM1_ESM/GAPDH (Figure4 C).jpg]

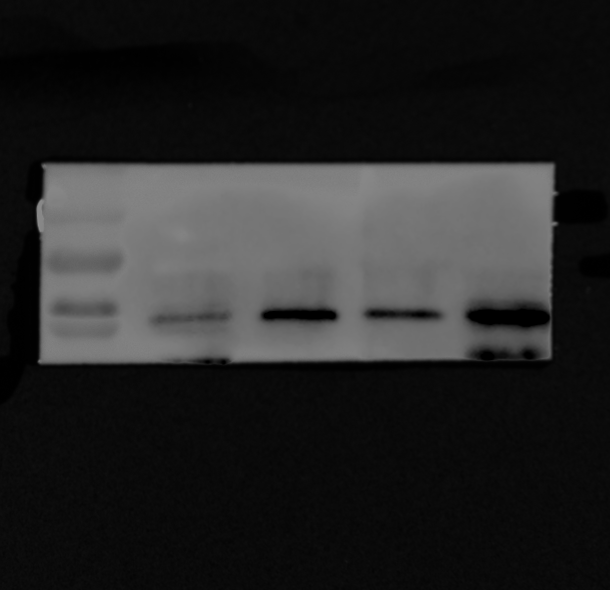

Supplement: Supplementary file 1 — Additional file 1. Figure S1. Flow chart. Figure S2. Expression of apoptosis-related factors. [file 12872_2022_2499_MOESM1_ESM.zip › 12872_2022_2499_MOESM1_ESM/12872_2022_2499_MOESM1_ESM/revised-Bax.tif]

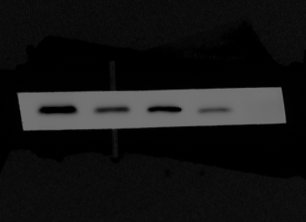

Supplement: Supplementary file 1 — Additional file 1. Figure S1. Flow chart. Figure S2. Expression of apoptosis-related factors. [file 12872_2022_2499_MOESM1_ESM.zip › 12872_2022_2499_MOESM1_ESM/12872_2022_2499_MOESM1_ESM/revised-Bcl-2+.tif]

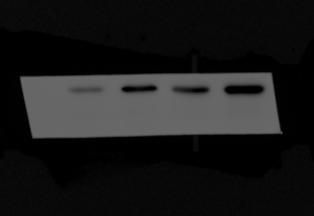

Supplement: Supplementary file 1 — Additional file 1. Figure S1. Flow chart. Figure S2. Expression of apoptosis-related factors. [file 12872_2022_2499_MOESM1_ESM.zip › 12872_2022_2499_MOESM1_ESM/12872_2022_2499_MOESM1_ESM/revised-Caspase3+.tif]

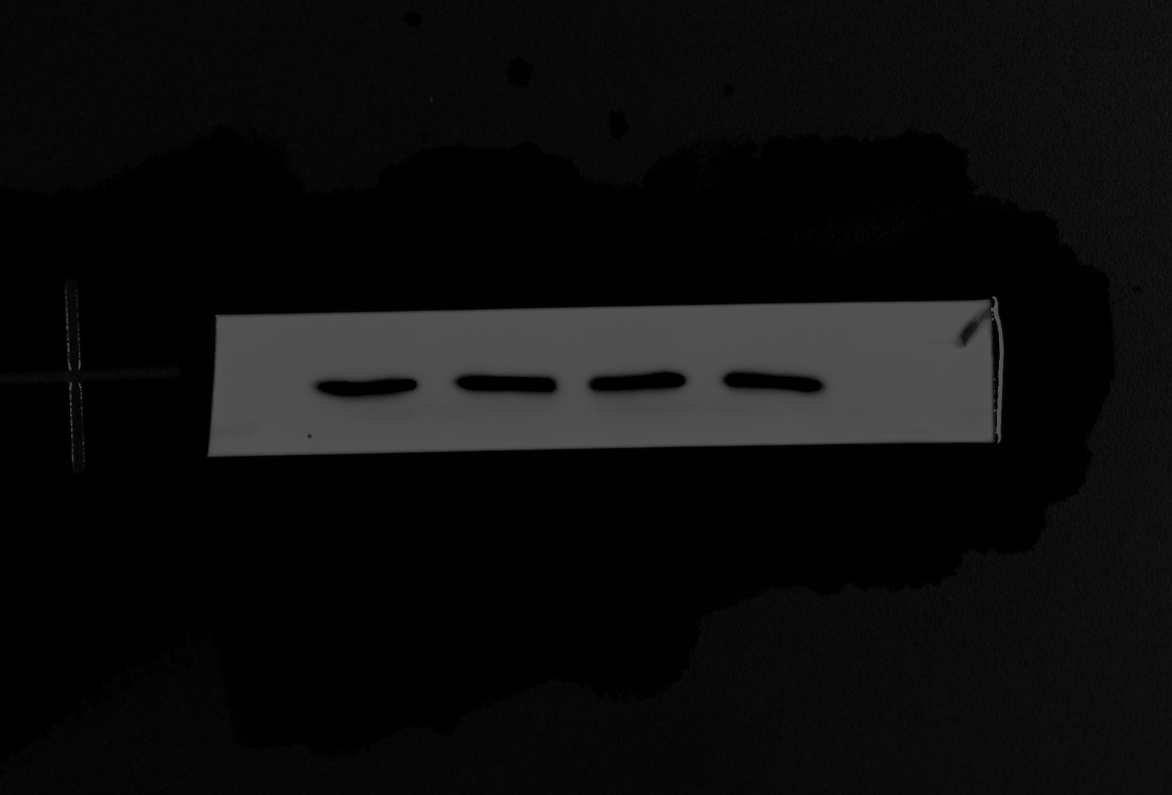

Supplement: Supplementary file 1 — Additional file 1. Figure S1. Flow chart. Figure S2. Expression of apoptosis-related factors. [file 12872_2022_2499_MOESM1_ESM.zip › 12872_2022_2499_MOESM1_ESM/12872_2022_2499_MOESM1_ESM/revised-GAPDH (FigureS2 A).tif]

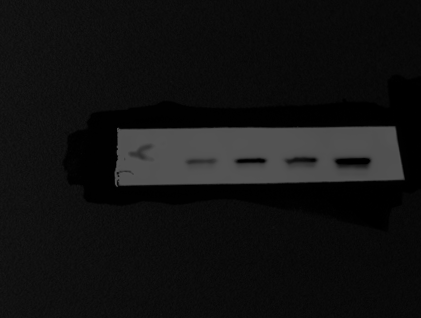

Supplement: Supplementary file 1 — Additional file 1. Figure S1. Flow chart. Figure S2. Expression of apoptosis-related factors. [file 12872_2022_2499_MOESM1_ESM.zip › 12872_2022_2499_MOESM1_ESM/12872_2022_2499_MOESM1_ESM/revised-P53+.tif]

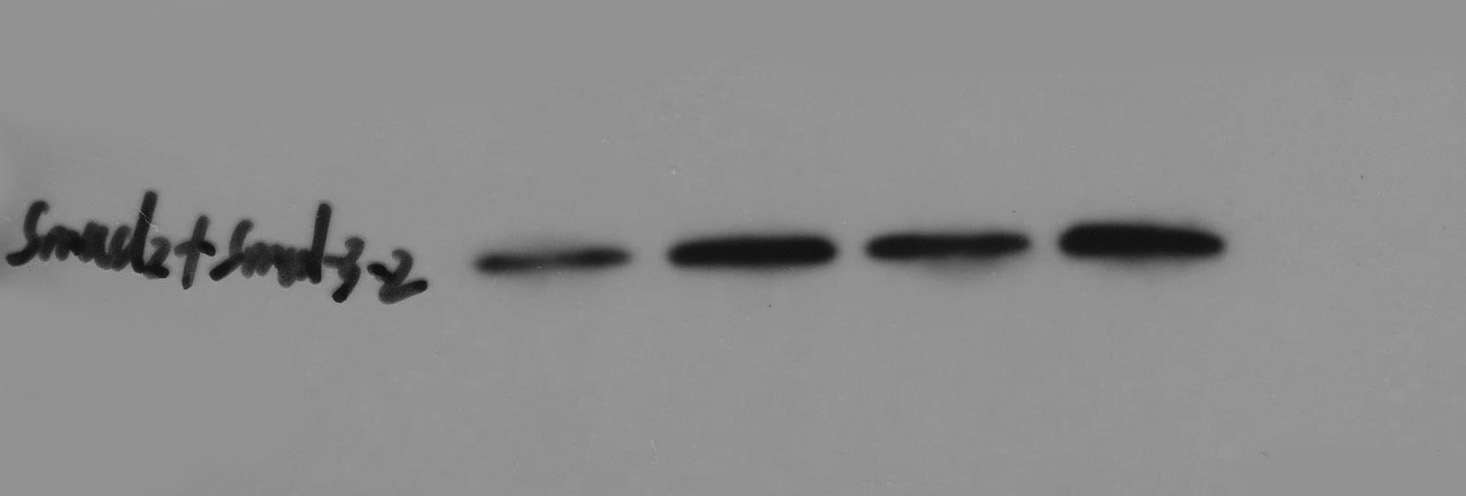

Supplement: Supplementary file 1 — Additional file 1. Figure S1. Flow chart. Figure S2. Expression of apoptosis-related factors. [file 12872_2022_2499_MOESM1_ESM.zip › 12872_2022_2499_MOESM1_ESM/12872_2022_2499_MOESM1_ESM/smad2+smad3-2.jpg]

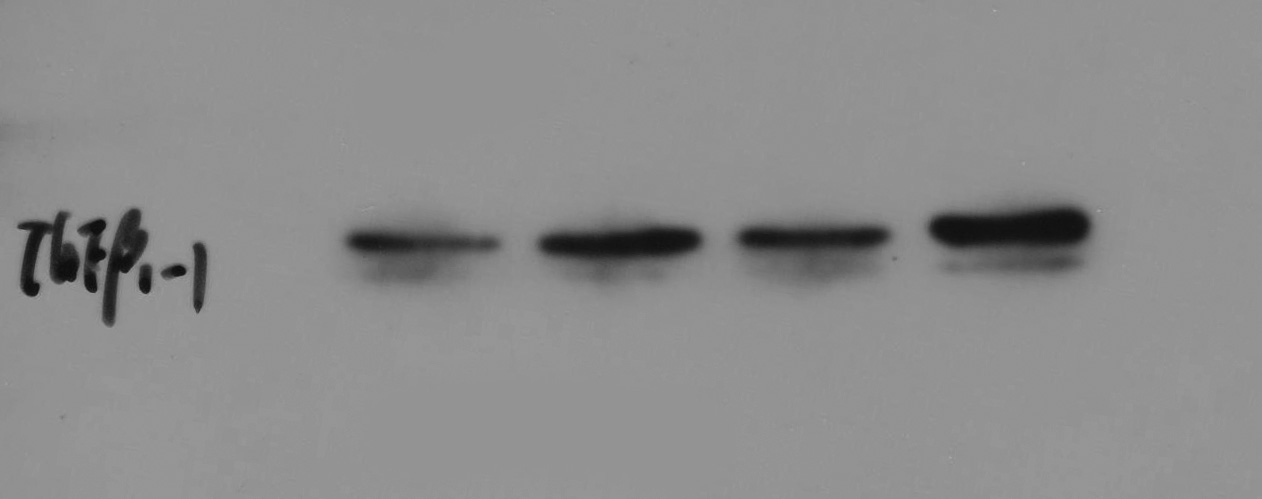

Supplement: Supplementary file 1 — Additional file 1. Figure S1. Flow chart. Figure S2. Expression of apoptosis-related factors. [file 12872_2022_2499_MOESM1_ESM.zip › 12872_2022_2499_MOESM1_ESM/12872_2022_2499_MOESM1_ESM/TGF1-1.jpg]
